# 

**Published:** 1890-04

**Authors:** 


### SPECIAL.

The Journal, in order to go forward in the best way, requires that its subscribers should be prompt in remitting the amount of their annual dues.

We shall feel greatly obliged to those in arrears if, without further notice, they will comply with this request, by sending the amount by postal order or otherwise, directly to the Journal's office.

Look over our list of premiums and make selections of what is most desired. Some of them are worth more than the price of a year's subscription.

### LITERARY.

A Galaxy of Progressive Poems.—Boston, Colby & Rich, Publishers. Any person who has been accustomed to visit the editorial sanctum of the *Banner of Light*, has doubtless been made acquainted with a little side office, just large enough to hold a desk and chair and divers shelvings for odds and ends, patched on to the walls at every place ample enough to hold a cigar box. He too has found in the chair fronting the desk, whose pigeon holes are stuffed to overflowing with documents of every conceivable shape, in a painful regularity of confusion, a middle aged, kindly spoken gentleman, wearing a green front-piece over his eyes, who takes your proffered hand with a hearty grasp, and a good natured smile that sets off the dingy sanctum like a gleam of sunshine in a cavern. This man is John W. Day, the assistant editor, who has been by turns printer, embryo clergyman, warrior and poet, and who, day in and day out, as we have means to know, as the one reliable always-on-hand and at-your-service adjunct of the good old *Banner*.

"Hath trod th' arena's floor
Where uses stern to beings call respond."

How one with such an eternal round of drudgery could find opportunity to coquette with the muses and strike to tuneful sound his responsive heart strings is a wonder, but so it is, as the handy little galaxy of poems attests, which the friends of progress will be glad to welcome to their firesides. Listen to him a moment:

"All souls in being's twilight track the vale
Where time's swift river seeks th' eternal sea;
Some dogma-laden walk with steps that fail,
Some with the stride of him whom truth makes free;
The cave-brewed Soma of man's earliest line
In schemes and forms diverse has flowed for him,
*But we this hour may drink the spirit wine*
*Whose currents need no creed's supporting brim.*"

---

Rothermel, a Story of Lost Identity.—American News Company, New York, Publishers' Agents. This is a story of unusual merit. The scene is laid principally in Boston, Paris and vicinity, during the late Franco-German war, with French and American characters and customs with which the author shows an intimate acquaintance. The versatile French demoiselle and the less demonstrable Boston girl are portrayed with the skill of the true artist, while some interesting phenomena are ingeniously wrought into the narrative, which proceeds without halting to the end. Price, 50 cents.

---

How to Preserve Health, by Louis Barker, M.D.; Exchange Printing Company, 47 Broad street, New York city, 1890, pp. 338. This is a manual of hygiene for both sexes, which extends from infancy to old age, and treats of almost every ailment that flesh is heir to in a plain, straightforward manner, without the use of technical words and obscure phrases, hence it is admirably adapted for a family guide. The subjects are well arranged, and if the advice given for preventing the inroads of disease are followed, there will be little need of calling in the family doctor. Price $1.

---

PEANUTS.—Three million two hundred thousand bushels of peanuts are consumed in this country every year. They come chiefly from Virginia and North Carolina, although Tennessee also produces a small crop. Peanuts are planted at corn planting time. Each kernel produces a running vine, like crab grass, and each root produces about twenty pods. When ripe, the plow is run through the loamy soil on a dry day, just before frost. The nuts are dried and shocked up like corn to keep dry before housing. When marketed, they go to a cleaner, where they are put through steam power machines and polished, after which they are graded according to size and variety. This year there is but two-thirds of a crop, and they are higher in price than since 1884. The crop begins to come into the market about the first of September. The Virginia nut is the largest and finest. The Wilmington is a smaller sort, and the Spanish nut, a still smaller variety, is one whose kernels peel perfectly clean, thus making it valuable for confectionary.

---

### THE WATER LILY.

The summer morning opens cool,
  A subtle freshness fills the air ;
And see ! upon the cloistered pool,
  The lily opes her bosom there.

Of all the buds and blossoms rare,
  No fairer one the eye may bless ;
She feels the zephyr's kindly care,
  And trembles at his fond caress.

Through all the loathsome mud and slime,
  She sends her roots to search below,
And undreamed beauties upward climb,
  And in her petals throb and glow.

Send down thy rootlets, O my soul !
  With darkened lives thy sunlight share,
And seek in miry depth and shoal,
  God's beauteous image buried there.

So, in some fair diviner hour,
  When risen free from sin and crime,
Thou shalt preserve life's perfect power
  Above the sluggish pools of time.

JOHN FRANKLIN KELLY, *in Christian Register.*

